# Supplementary figures and images for: RNA-Sequencing Based microRNA Expression Signature of Colorectal Cancer: The Impact of Oncogenic Targets Regulated by miR-490-3p
Source: Int J Mol Sci. 2021 Sep 13;22(18):9876. doi: 10.3390/ijms22189876 (PMC8469425; doi:10.3390/ijms22189876)

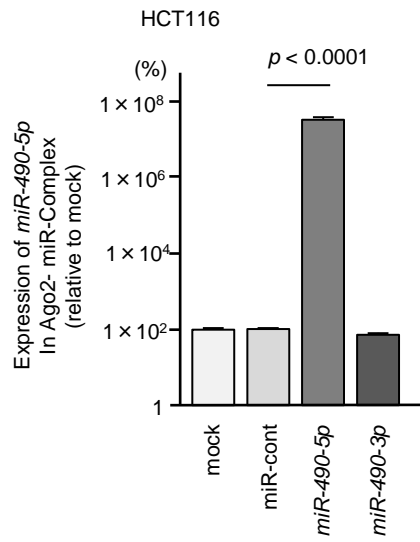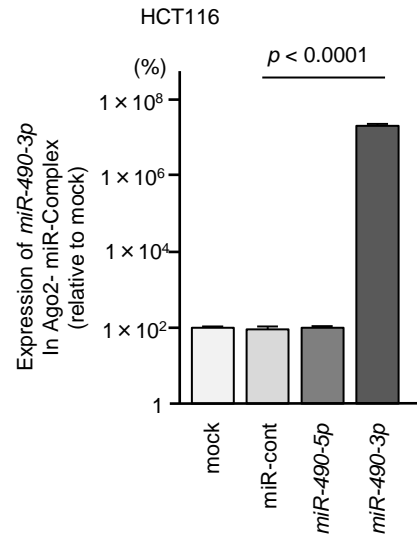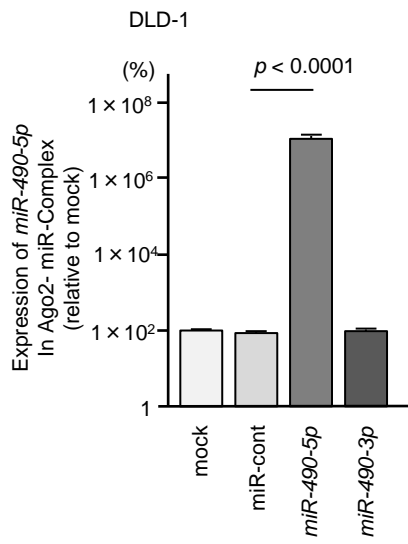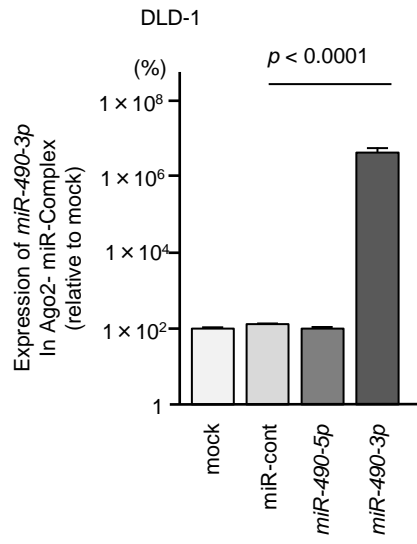

Supplement: Supplementary file 1 [file ijms-22-09876-s001.zip › Figures S1.pdf]
